# Supplementary material for: A roadmap from the bond strength to the grain-boundary energies and macro strength of metals
Source: Nat Commun. 2025 Jan 13;16:615. doi: 10.1038/s41467-025-55921-y (PMC11731018; doi:10.1038/s41467-025-55921-y)
Supplement: Supplementary file 1 — Supplementary Information [file 41467_2025_55921_MOESM1_ESM.pdf]

Supplementary Information

A Roadmap from the Bond Strength to the Grain-Boundary Energies and Macro Strength of Metals

Xin Li[1], Hao Wu[1], Wang Gao[1,*], and Qing Jiang[1]

[1]Key Laboratory of Automobile Materials, Ministry of Education, Department of Materials Science and Engineering, Jilin University, 130022, Changchun, China

*wgao@jlu.edu.cn

Supplementary Figure 1. 2
Supplementary Figure 2. 3
Supplementary Figure 3. 4
Supplementary Figure 4. 5
Supplementary Figure 5. 6
Supplementary Figure 6. 7
Supplementary Figure 7. 8
Supplementary Figure 8. 9
Supplementary Table 1. 10
Supplementary Table 2. 11
References 12

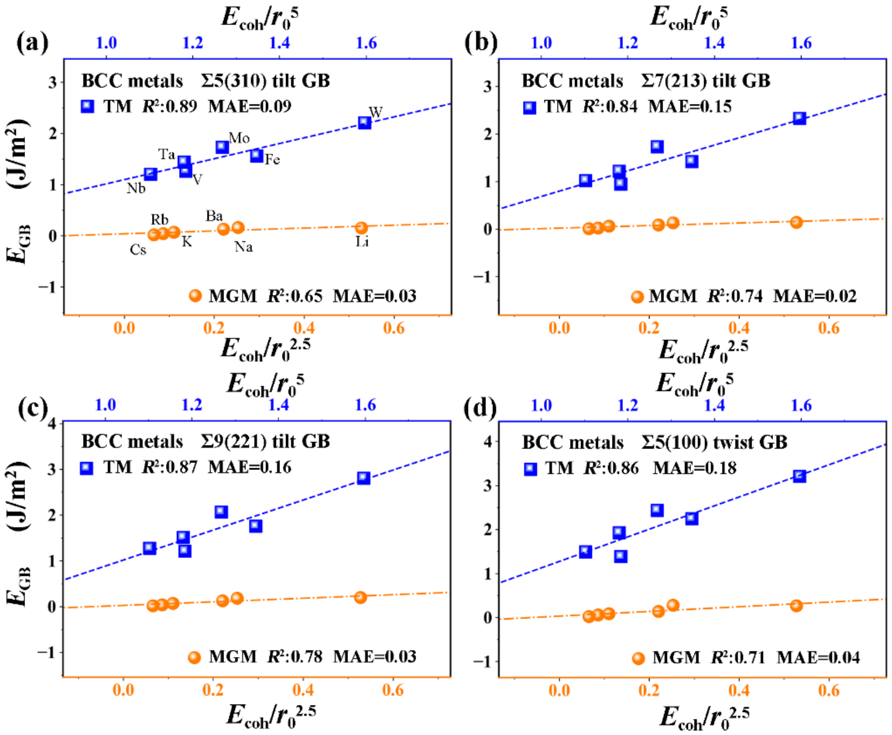

Supplementary Figure 1. Grain-boundary (GB) energies as the functions of our descriptors for different body-centered-cubic (BCC) GBs. (a) Σ5(310), (b) Σ7(213), (c) Σ9(221) and (d) Σ5(100) twist GBs[1]. The blue squares and orange dots represent transition metals (TMs) and main-group metals (MGMs). The accuracy is measured by mean absolute error (MAE) and regression coefficient (R^2). All the dashed lines are obtained from linear fitting. The corresponding metal composition of each datapoint can be found in the source data file. Source data are provided as a Source Data file.

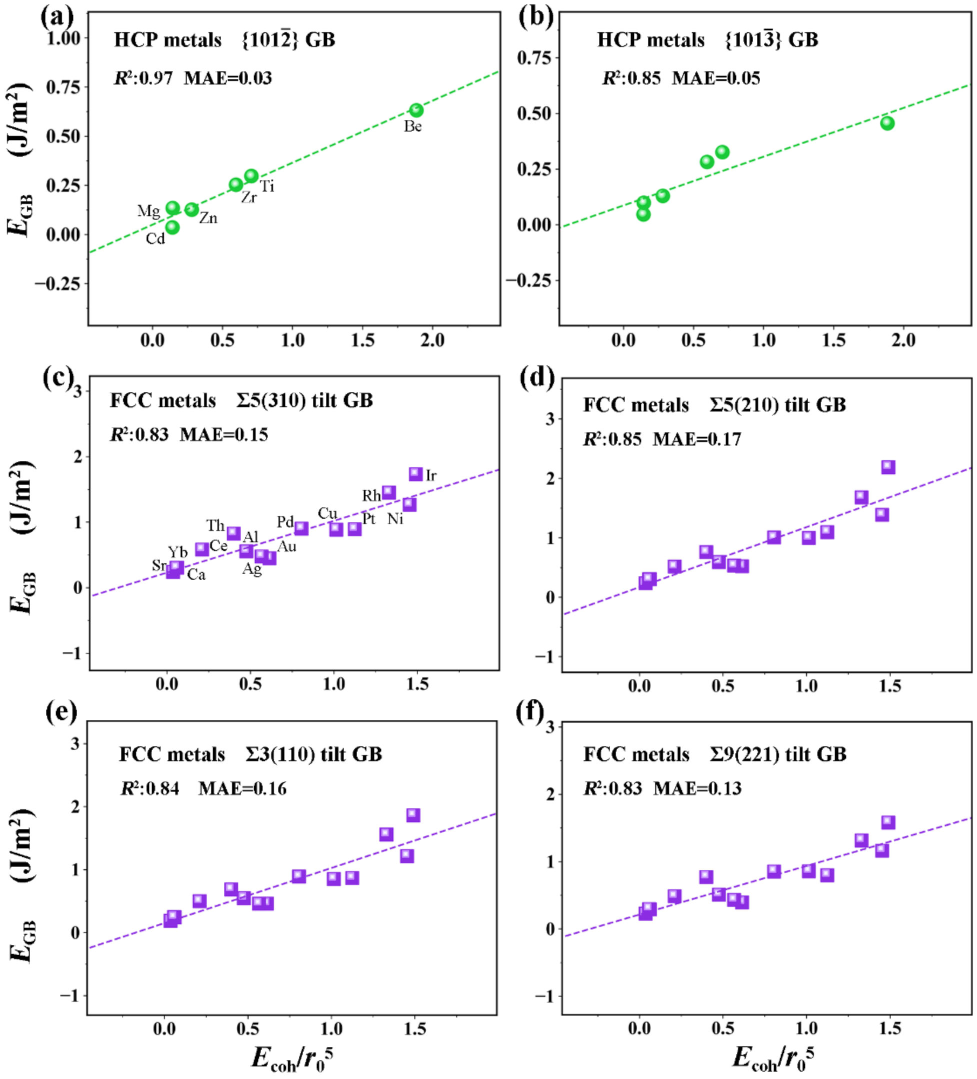

Supplementary Figure 2. Grain-boundary (GB) energies as the functions of our descriptors for different hexagonal-close-packed (HCP) and face-centered-cubic (FCC) GBs. (a) {10$\bar{1}$2} and (b) {10$\bar{1}$3} HCP GBs, (c) Σ5(310), (d) Σ5(210), (e) Σ3(110) and (f) Σ9(221) FCC GBs[1]. The green dots and purple squares represent HCP and FCC metals respectively. The accuracy is measured by mean absolute error (MAE) and regression coefficient (R^2). All the dashed lines are obtained from linear fitting. The corresponding metal composition of each datapoint can be found in the source data file. Source data are provided as a Source Data file.

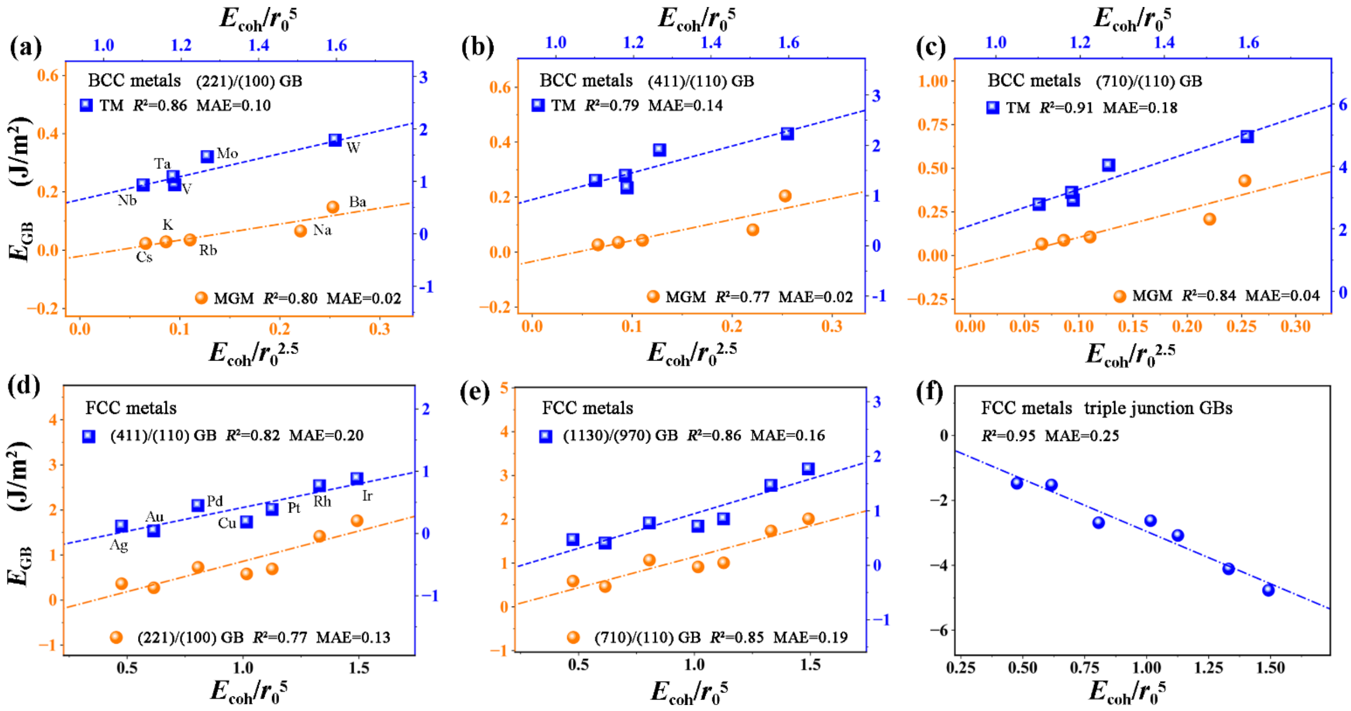

Supplementary Figure 3. Grain-boundary (GB) energies as the functions of our descriptors for complex asymmetric GBs. (a) (221)/(110), (b) (411)/(100), and (c) (710)/(110) body-centered-cubic (BCC) GBs, (d) (221)/(110), (411)/(100), (e) (710)/(110), and (1130)/(970) face-centered-cubic (FCC) GBs, and (f) the triple junction of FCC GBs with Σ3(111)-Σ3(111)-Σ9(221). The accuracy is measured by mean absolute error (MAE) and regression coefficient (R^2). All the dashed lines are obtained from linear fitting. The corresponding metal composition of each datapoint can be found in the Source Data file. Source data are provided as a Source Data file.

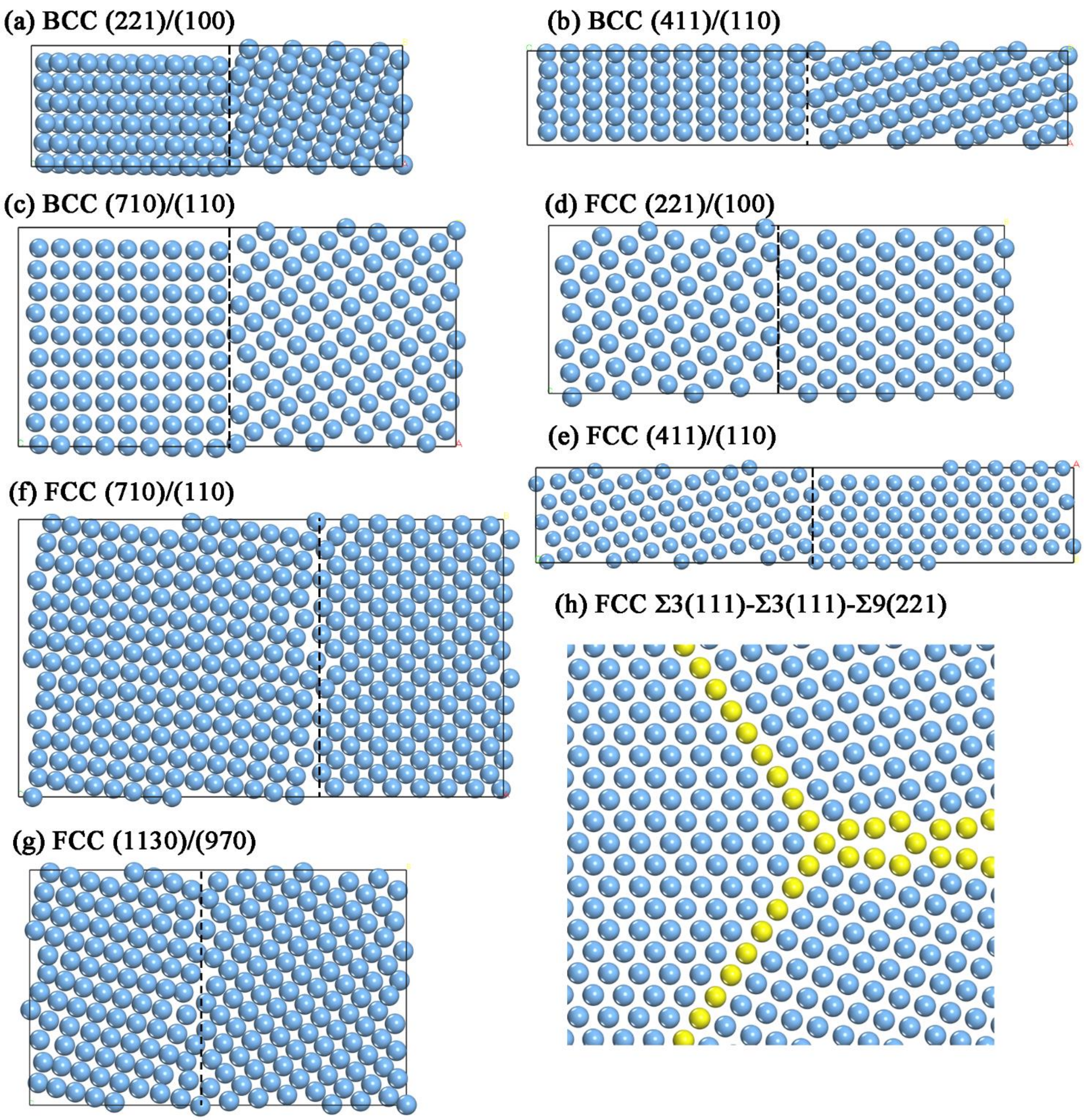

Supplementary Figure 4. The structures of asymmetric grain boundaries (GBs). (a) (221)/(110), (b) (411)/(100) and (c) (710)/(110) body-centered-cubic (BCC) GBs, and (d) (221)/(110), (e) (411)/(100), (f) (710)/(110) and (g) (1130)/(970) face-centered-cubic (FCC) GBs, and (h) the triple junction FCC GBs of Σ3(111)-Σ3(111)-Σ9(221). The yellow atoms are located at GBs. The atomic positions are provided in the Source Data file.

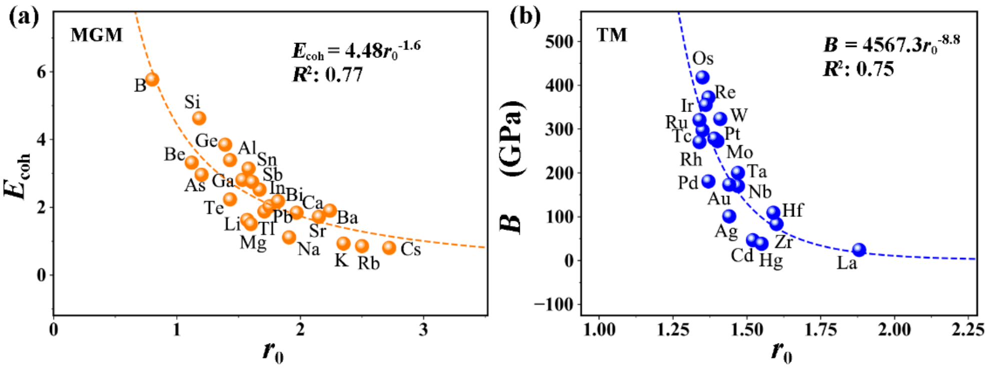

Supplementary Figure 5. The comparison between our framework and classical bulk-modulus theories. (a) The cohesive energies E_{coh} of main-group elements[2] (MGMs, orange dots) and the (b) bulk modulus B of transition metals[3] (TMs, blue dots) as a function of the atomic radius r_0. The accuracy is measured by regression coefficient (R^2). All the dashed lines are obtained from fitting. Source data are provided as a Source Data file.

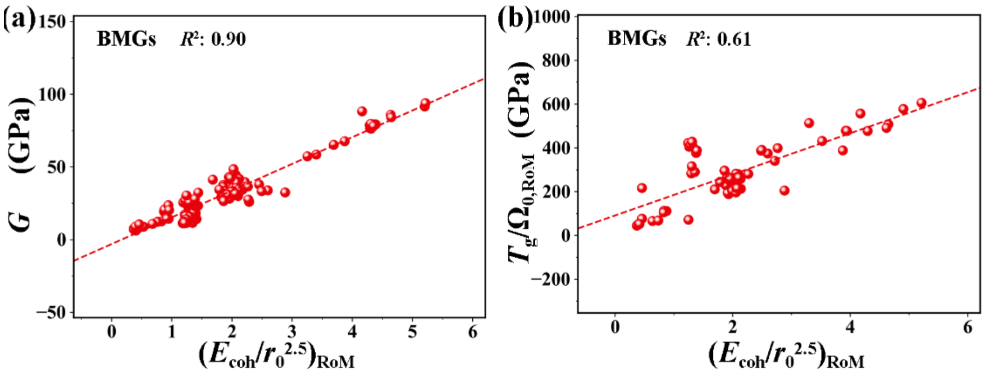

Supplementary Figure 6. Macro properties of bulk metallic glasses (BMGs) as the functions of our descriptors[4]. The correlation between (a) shear modulus G with our descriptor, and (b) $T_g/\Omega_{0,RoM}$ with our descriptor. The accuracy is measured by regression coefficient (R^2). All the dashed lines are obtained from linear fitting. The corresponding alloy composition of each datapoint can be found in the Source Data file.

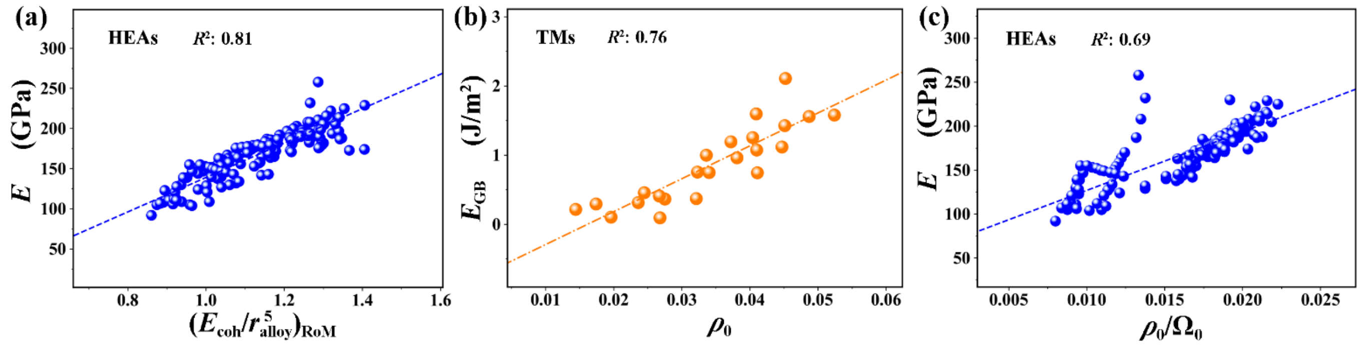

Supplementary Figure 7. Comparison between our framework with the previous descriptors. (a) The relationship between Young's modulus of high-entropy alloys (HEAs) with our descriptor $(E_{coh}/r_{alloy}{}^5)_{RoM}$. The radii r_{alloy} are the size of the elements in alloy phase. (b) The correlation between GB energies of the transition metals (TMs) and the interstitial electronic density ρ_0. (c) The correlation between Young's modulus of HEAs and the descriptor ρ_0/Ω_0. The accuracy is measured by regression coefficient (R^2). All the dashed lines are obtained from linear fitting. The corresponding metal and alloy composition of each datapoint can be found in the Source Data file.

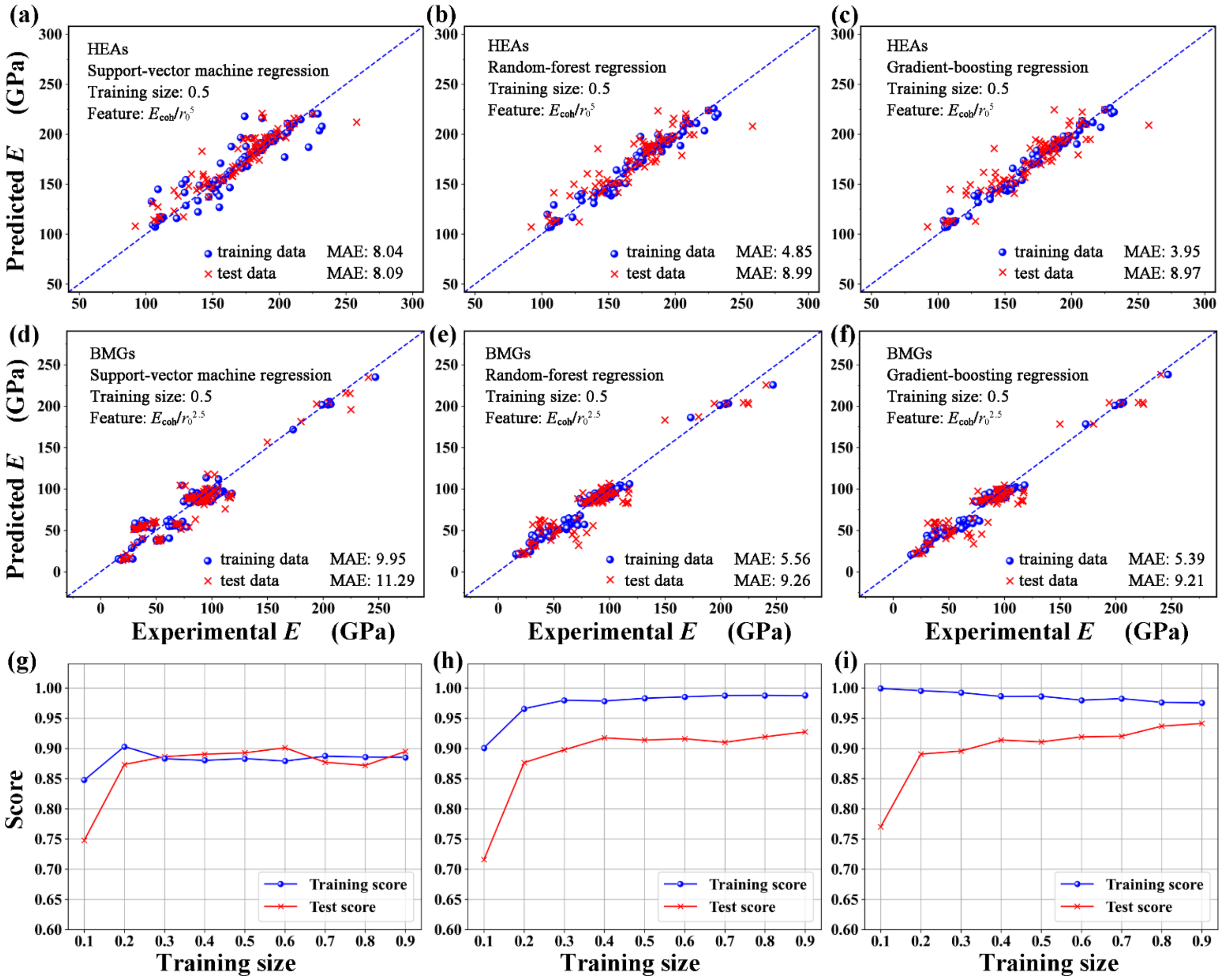

Supplementary Figure 8. Machine learning framework combining with our descriptors in determining the Young's modulus E of high-entropy alloys (HEAs) and bulk metallic glasses (BMGs). (a-c) Machine learning models combined with E_{coh}/r_0^5. (a) Support-vector machine, (b) random-forest regression, and (c) gradient boosting decision tree algorithm. (d-f) Machine learning models combined with $E_{coh}/r_0^{2.5}$. (d) Support-vector machine, (e) random-forest regression, and (f) gradient boosting decision tree algorithm. (g) Testing curve of support-vector machine models in HEAs, (h) random-forest regression and (i) gradient boosting decision tree models in BMGs. The accuracy is measured by mean-absolute error (MAE) and the regression score. All the blue dashed lines in (a-f) indicate that the predicted modulus is equal to the experimental ones. The corresponding alloy composition of each datapoint can be found in the Source Data file.

Supplementary Table 1. Summary of cohesive energies E_{coh} and atomic radii r_0 for the transition metals, main-group elements and the rare-earth elements.

metals	E_{coh}	r_0	metals	E_{coh}	r_0
Sc	3.90	1.64	Ir	6.94	1.36
Ti	4.85	1.47	Pt	5.84	1.39
V	5.31	1.35	Au	3.81	1.44
Cr	4.10	1.29	Hg	0.67	1.55
Mn	2.92	1.37	Li	1.63	1.57
Fe	4.28	1.26	Be	3.32	1.12
Co	4.39	1.25	B	5.77	0.80
Ni	4.44	1.25	Na	1.11	1.91
Cu	3.49	1.28	Mg	1.51	1.60
Zn	1.35	1.37	Al	3.39	1.43
Y	4.37	1.82	Si	4.63	1.18
Zr	6.25	1.60	K	0.93	2.35
Nb	7.57	1.47	Ca	1.84	1.97
Mo	6.82	1.40	Rb	0.85	2.50
Tc	6.85	1.35	Sr	1.72	2.15
Ru	6.74	1.34	Cs	0.80	2.72
Rh	5.75	1.34	Ba	1.90	2.24
Pd	3.89	1.37	Pb	2.03	1.75
Ag	2.95	1.44	Ce	4.32	1.83
Cd	1.16	1.52	Pr	3.70	1.82
La	4.47	1.88	Dy	3.04	1.75
Hf	6.44	1.59	Ho	3.14	1.74
Ta	8.10	1.47	Er	3.29	1.73
W	8.90	1.41	Yb	1.60	1.94
Re	8.03	1.37	C	7.37	0.77
Os	8.17	1.35	Si	4.63	1.18

Supplementary Table 2. The GB planes, lattices, layers, number of atoms, lattice parameters and k-points for the studied asymmetric GBs. Here a_0 is the lattice parameters of the bulk structures.

GBs	Lattice	Layers of grain 1	Layers of grain 2	Number of atoms	Lattice parameters	k-points
(221)+(110)	FCC	10	12	276	$4.24a_0$,$1.41a_0$, $11.50a_0$	6×2×1
(411)+(100)	FCC	14	14	284	$1.41a_0$, $3a_0$, $16.97a_0$	8×4×1
(710)+(110)	FCC	7	8	340	a_0, $7.07a_0$, $12.35a_0$	10×2×1
(1310)+(110)	FCC	9	7	208	a_0, $5.70a_0$, $9.12a_0$	10×2×1
(221)+(110)	BCC	10	12	152	$4.24a_0$,$1.41a_0$, $13.00a_0$	8×4×1
(411)+(100)	BCC	16	12	142	$1.41a_0$, $3a_0$, $17.19a_0$	8×4×1
(710)+(110)	BCC	7	9	192	a_0, $7.07a_0$, $14.12a_0$	12×2×1

References

1. Zheng, H. et al. Grain boundary properties of elemental metals. *Acta Mater.* **186**, 40-49 (2020).
2. Kittel, C. *Introduction to Solid State Physics*. 8th edition edn (Hoboken, NJ: John Wiley & Sons, Inc, 2005).
3. Lide, D. R. *CRC Handbook of Chemistry and Physics*. 88th edition edn (Boca Raton, Florida: Taylor & Francis Group, 2008).
4. Wang, W. H. The elastic properties, elastic models and elastic perspectives of metallic glasses. *Prog. Mater. Sci.* **57**, 487-488 (2012).
